# The use of the temporal scan statistic to detect methicillin-resistant *Staphylococcus aureus* clusters in a community hospital

**DOI:** 10.1186/1471-2334-14-375

**Published:** 2014-07-08

**Authors:** Meredith C Faires, David L Pearl, William A Ciccotelli, Olaf Berke, Richard J Reid-Smith, J Scott Weese

**Affiliations:** 1 Department of Population Medicine, University of Guelph, Guelph, Ontario, Canada; 2 Infection Prevention and Control, Grand River Hospital, Kitchener, Ontario, Canada; 3 Department of Pathology and Molecular Medicine, McMaster University, Hamilton, Ontario, Canada; 4 Department of Mathematics and Statistics, University of Guelph, Guelph, Ontario, Canada; 5 Department of Pathobiology, University of Guelph, Guelph, Ontario, Canada

**Keywords:** Methicillin-resistant *Staphylococcus aureus*, Clusters, Temporal scan statistic, Community hospital, Epidemiology, *Spa* typing

## Abstract

**Background:**

In healthcare facilities, conventional surveillance techniques using rule-based guidelines may result in under- or over-reporting of methicillin-resistant *Staphylococcus aureus* (MRSA) outbreaks, as these guidelines are generally unvalidated. The objectives of this study were to investigate the utility of the temporal scan statistic for detecting MRSA clusters, validate clusters using molecular techniques and hospital records, and determine significant differences in the rate of MRSA cases using regression models.

**Methods:**

Patients admitted to a community hospital between August 2006 and February 2011, and identified with MRSA > 48 hours following hospital admission, were included in this study. Between March 2010 and February 2011, MRSA specimens were obtained for *spa* typing. MRSA clusters were investigated using a retrospective temporal scan statistic. Tests were conducted on a monthly scale and significant clusters were compared to MRSA outbreaks identified by hospital personnel. Associations between the rate of MRSA cases and the variables year, month, and season were investigated using a negative binomial regression model.

**Results:**

During the study period, 735 MRSA cases were identified and 167 MRSA isolates were *spa* typed. Nine different *spa* types were identified with *spa* type 2/t002 (88.6%) the most prevalent. The temporal scan statistic identified significant MRSA clusters at the hospital (n = 2), service (n = 16), and ward (n = 10) levels (P ≤ 0.05). Seven clusters were concordant with nine MRSA outbreaks identified by hospital staff. For the remaining clusters, seven events may have been equivalent to true outbreaks and six clusters demonstrated possible transmission events. The regression analysis indicated years 2009–2011, compared to 2006, and months March and April, compared to January, were associated with an increase in the rate of MRSA cases (P ≤ 0.05).

**Conclusions:**

The application of the temporal scan statistic identified several MRSA clusters that were not detected by hospital personnel. The identification of specific years and months with increased MRSA rates may be attributable to several hospital level factors including the presence of other pathogens. Within hospitals, the incorporation of the temporal scan statistic to standard surveillance techniques is a valuable tool for healthcare workers to evaluate surveillance strategies and aid in the identification of MRSA clusters.

## Background

In the healthcare setting, the timely identification of healthcare-associated infections (HAIs) and the institution of infection control measures are crucial steps for the prevention and reduction of transmission events and outbreaks in the patient population. However, the detection of transmission events is based on limited evidence [1], and detection of specific pathogen clusters is generally subjective in nature [2]. Furthermore, even well-established infection prevention strategies can be often disregarded [3] at various levels. Overall, these factors may lead to the transmission of pathogens within the hospital, a missed opportunity to investigate a disease cluster, or false ascertainment of a cluster resulting in the misuse of hospital resources for investigational purposes [2]. Statistical methods, such as the scan statistic, may enhance the identification of disease clusters and/or outbreaks in the hospital setting [2,4]. The scan statistic [5] can be used to detect and evaluate clusters of infectious diseases in space, time, and/or space-time [6-8]. By understanding the clustering of infectious diseases spatially and/or temporally, potential risk factors can be identified [9], detailed investigations determining the association between exposures and disease interventions can be facilitated [10], and outbreaks can be detected [6].

Methicillin-resistant *Staphylococcus aureus* (MRSA) outbreaks in healthcare facilities represent a significant burden to public health in terms of ward closures, infection control measures, increased patient morbidity and mortality rates, and healthcare costs [11,12]. In hospitals, identification of outbreaks is routinely based on the examination of microbiological test results and patients’ charts [4], with the definition of an outbreak generally relying on rule-based criteria [2]. However, these types of criteria are prone to error since they fail to address changes in population size or random variation [2]. With the increasing availability of timely surveillance data within the hospital setting, the use of analytical methods may lead to the earlier detection of disease clusters or outbreaks [13]. In hospitals, the incorporation of the scan statistic for the detection of MRSA disease clusters, spatially and/or temporally, has been limited. In one study, a space-time permutation scan statistic was utilized to detect clusters throughout the hospital [2]. However, molecular data were not collected to validate MRSA clusters. In another investigation, a temporal scan statistic, incorporating molecular data, was employed to identify MRSA clusters which were used as a gold standard to evaluate other algorithms for cluster detection [4]. For both investigations, the application of a scan statistic to hospital data resulted in the identification of several significant MRSA clusters that were not identified by hospital personnel. However, as both studies were conducted in academic medical centres, further investigation of the scan statistic for identifying MRSA clusters under different healthcare settings is required [2,4].

The objectives of this study were to investigate the utility of the temporal scan statistic for detecting MRSA clusters in a community hospital and to validate significant clusters using molecular techniques and hospital records concerning known MRSA outbreaks; and to determine if there were significant differences in the rate of MRSA infection and colonization cases by month, season, and year using regression models.

## Methods

### Study site

Located in southern Ontario, Canada, the participating study hospital has 345 beds, over 200,000 in- and out-patient visits annually, and provides an array of services including internal medicine, surgery, emergency, pediatrics, oncology, rehabilitation, intensive care, and psychiatry. Both urban and rural populations are served by this facility. This study was approved by the research ethics boards of the University of Guelph and the participating hospital. The research ethics approval covered all aspects of the study including the collection of de-identified isolates from the hospital’s microbiology laboratory.

### Surveillance

In the study hospital, targeted surveillance for MRSA is conducted based on recommendations provided by a Provincial Infectious Diseases Advisory Committee [14]. Briefly, during the study period, at the time of hospital admission, patients identified as having an increased risk for MRSA acquisition are screened. These risk factors include [14]:

• Previous colonization or infection with MRSA;

• Time spent in any healthcare facility in the previous twelve months (time defined as > 12 continuous hours);

• Recent exposure to a unit/ward/area of a healthcare facility identified with an MRSA outbreak; or

• Individuals receiving home healthcare or treatment with an indwelling medical device.

For MRSA detection, specimens are obtained from the anterior nares and the perineal area (surveillance sample); and from any skin lesions, wounds, incisions, ulcers, and exit sites of indwelling devices if present (surveillance and/or clinical sample) [14].

During an outbreak or for patients located on high risk units (e.g., intensive care unit), universal admission screening is conducted. For patients hospitalized for an extended period of time, weekly MRSA surveillance cultures are performed. In addition, for patients identified with indwelling medical devices or wounds, these sites are monitored by weekly culture. If a patient is identified as an MRSA contact (i.e., roommate of a patient who is found to be MRSA positive), the MRSA contact will have two sets of surveillance cultures taken with at least one set taken seven days following their last exposure to the MRSA patient [14]. In the participating hospital, MRSA cases were classified as healthcare-associated (HA) if the patient was newly identified with MRSA (infection or colonization) >48 hours following hospital admission.

### Patient data and isolate collection from the hospital

For a case to be included in this investigation, MRSA was newly identified between August 1, 2006 and February 28, 2011 and > 48 hours following hospital admission. Patients that were identified as being either infected or colonized with MRSA were included. Only one MRSA isolation event per patient per admission-discharge period was included in the analyses. The admission-discharge period was defined as the time interval from when a patient was admitted to, and discharged from, the hospital. Transfer to another ward within the hospital was not considered a discharge. For a patient to be admitted ≥ 2 times to the hospital, > 24 hours between the discharge and admission dates was required. Data from the first bacteriology report per patient per admission-discharge period were obtained. Bacteriology reports from MRSA cases located in the emergency and hemodialysis wards were excluded as these departments specifically support outpatients.

For MRSA cases identified between March 1, 2010 and February 28, 2011, the hospital’s microbiology laboratory collected and submitted patient MRSA isolates for molecular typing. In the participating facility, MRSA was confirmed from surveillance samples using chromogenic agar (BBL CHROMagar, MRSA, Becton, Dickinson and Company, Sparks, Maryland, USA), the penicillin-binding protein 2a latex agglutination test (MRSA latex agglutination test, Oxoid Ltd., Hants, UK), and Vitek^®^ 2 (bioMérieux, Marcy-l’Étolie, France) according to the manufacturers’ instructions. For clinical samples, *S. aureus* was confirmed using the gram stain and the tube coagulase test and MRSA was confirmed using Vitek^®^ 2. For surveillance and clinical MRSA isolates, a standard panel of antimicrobials was used for susceptibility testing and included rifampin, clindamycin, trimethoprim and sulfamethoxazole, and vancomycin. Testing was performed using Vitek^®^ 2 and results were based on the standards set by the Clinical and Laboratory Standards Institute [15]. At the hospital level, all specimens submitted for MRSA testing were collected at the discretion of medical personnel. Only one MRSA isolate per patient was collected for molecular typing.

Data collected from the bacteriology report included a unique patient identifier, dates pertaining to patient admission, discharge, and when a specimen was collected for MRSA testing, the ward location of the patient when the specimen was obtained, and the antimicrobial susceptibility profile of the MRSA isolate. For ward location, bacteriology reports provided both service and ward designations. Services were defined as specific departments (e.g., internal medicine, surgery) whereas wards were characterized as specific, physically distinct units that comprised a service (e.g., wards S1 and S2 constituted the surgery service).

Information pertaining to the number of patient days per month for each service was obtained. For wards, data on patient days were collected only from those wards that were operational and provided the same service for the complete study period (i.e., 55 months). For descriptive statistics, incidence rates for MRSA were expressed as the number of MRSA cases per 10,000 patient days.

Data pertaining to known MRSA outbreaks that occurred during the study period (e.g., start and end date, ward location, and number of patients involved) were collected from the hospital’s Infection Prevention and Control (IPC) department. The molecular identity of MRSA strains involved in previous outbreaks was not available as this healthcare facility does not routinely analyze MRSA strains at the molecular level. In the study hospital, standardized rule-based criteria to identify MRSA outbreaks are not employed. Due to the lack of a formal definition, MRSA outbreaks were identified by hospital personnel based on an increase in the baseline MRSA rate over a rapid time period, especially when localized to one patient care area.

### Molecular typing

Patient isolates were obtained from the hospital’s microbiology laboratory following MRSA confirmation. Isolates were collected from culture plates using a sterile culture swab with Stuart’s media and forwarded to the laboratory at the University of Guelph. Upon arrival, culture swabs were streaked onto blood agar (Oxoid, Nepean, Ontario, Canada) and incubated at 35°C, aerobically, for 24 hours. Colonies were identified as *S. aureus* by Gram stain, catalase test, tube coagulase test, and the *S. aureus* latex agglutination assay (Pastorex Staph-plus, Bio-Rad Laboratories Ltd., Mississauga, Ontario, Canada). The presence of methicillin-resistance was confirmed by the penicillin-binding protein 2a latex agglutination test (MRSA latex agglutination test, Oxoid Ltd., Hants, UK).

Molecular typing of MRSA was conducted using sequence analysis of the X region of the staphylococcal protein A gene (*spa* typing) [16]. Sequences were then analyzed using two different methodologies; eGenomics software [17] and the Ridom system [18]. Based on eGenomics, *spa* types are reported using a numerical system (e.g., *spa* type 2) whereas Ridom *spa* types are reported using a numerical system preceded by a ‘t’ (e.g., t002). The *spa* types were compared to epidemic MRSA strains that are frequently found in Canada [19]. In addition, all MRSA isolates were investigated for the *luk*F-PV gene encoding the Panton-Valentine leukocidin (PVL) toxin by real-time PCR [20].

To determine the clustering of *spa* types, all *spa* typing data were imported into BioNumerics (version 6.6; Applied Maths, Ghent, Belgium) and were analyzed using the *spa* typing plug-in tool. A minimum spanning tree (MST) was constructed using the default distance bin size of 100%. Only *spa* types that differed by ≤ 2 repeats were considered to be closely related [21].

### Statistical analysis

All bacteriology reports were provided by the hospital in electronic format. The temporal scan statistic was performed using SaTScan version 9.0 [5] and all descriptive statistics and model building were conducted using Stata 11.0 (StataCorp, College Station, Texas, USA). For all hypothesis tests, a 5% significance level was applied (α ≤ 0.05), if not stated otherwise.

### Statistical model

To evaluate the association between the rate of MRSA cases in the hospital and the independent variables year, month, and season, a Poisson regression analysis was conducted. For the independent variable season, months were categorized in the following groupings: winter (January – March), spring (April – June), summer (July – September), and fall (October – December). The dependent variable and offset were the number of MRSA cases and the natural log of the population at risk (i.e., patient days), respectively, for a particular month. Due to the hierarchical structure of the data, MRSA cases nested in wards, a multilevel Poisson model including a random intercept for ward and a fixed effect for service, was used to adjust for clustering. Specifically, the variable service was categorized as medicine (intensive care, oncology, pediatrics, internal medicine), surgery, and other (psychiatry, rehabilitation, hospice, childbirth, nursery).

The Spearman’s rank correlation coefficient was used to identify correlations between all independent variables. Variables with a correlation above 0.8 were investigated for collinearity and the biologically more plausible variable was retained in the model [22]. Univariable multilevel Poisson models were fit using marginal likelihood estimation via the adaptive quadrature algorithm (as implemented in the xtmepoisson procedure in Stata) to screen each independent variable with the dependent variable using a liberal significance level (α ≤ 0.20). Manual backwards step-wise modeling was applied to fit a multivariable multilevel Poisson model to all previously identified significant co-variables. To assess the significance of the independent variables, the likelihood ratio test was utilized. Confounding was evaluated by examining the effect of the removed variables on the coefficients of the remaining variables. A variable was considered to be a confounder if it changed the model coefficients by ≥ 20% [23]. Interaction terms were examined for all independent variables. Due to concerns regarding unexplained overdispersion, the Poisson random effects model was compared to a negative binomial random effects model using Akaike’s Information Criteria (AIC). The random effects negative binomial model allowed the overdispersion parameter to vary randomly by cluster based on a beta distribution (using the xtnbreg command in Stata) [24]. The model with the lowest AIC was selected. Based on the final multivariable model, contrasts for independent variables with >2 categories were examined to investigate significant differences between any two categories.

### Temporal scan statistic

To identify MRSA clusters, the temporal scan statistic employing a Poisson model was used. The scan statistic involves a flexible scanning window that gradually moves across time. The number of observed and expected observations inside the window is compared to outside the window, at each time period, with the greatest excess of observed cases noted [5,13]. Under the null hypothesis, the expected number of cases in each time period covered by the scanning window is proportional to its population size; whereas under the alternative hypothesis, there is an elevated risk within the window as compared to outside the window [5]. For this investigation, the population size was defined as the number of patient-days for each service and ward on a monthly basis. The window identified as least likely due to chance, is subsequently evaluated by a maximum likelihood test with a test decision based on a Monte-Carlo simulated P-value [5]. Monte Carlo replications were set at 9999 for this analysis.

To detect MRSA clusters, only periods with high rates (i.e., a one-tailed test) were scanned. The maximum temporal window size was set to 50% of the study period. In addition, the scan test was adjusted for more likely clusters via an iterative test procedure with the identified clusters from previous iterations removed from the data set and a new analysis performed using the remaining data [5]. Data were analyzed on a monthly scale. A cluster was defined as a period where the rate of MRSA cases was statistically higher than expected inside a window compared to outside a window.

Retrospective monthly scan tests were conducted for the entire study period (i.e., August, 2006 to February, 2011) as well as annually (January 1^st^ – December 31^st^) from 2006 to 2011. For 2006, the time interval was restricted to August 1^st^ – December 31^st^ and for 2011, the time interval was limited to January 1^st^ – February 28^th^. For each time interval, temporal scan tests were conducted to identify MRSA clusters at three different levels including hospital wide, services, and wards. For this analysis, 10 services were identified and included acute care, complex care, hospice, the intensive care unit, internal medicine, oncology, pediatrics, psychiatry, rehabilitation, and surgery. Five wards were identified and included M1 (internal medicine), S1 (surgery), S2 (surgery), C1 (complex care), and C2 (complex care).

Significant (P ≤ 0.05) clusters that were identified by the temporal scan statistic were compared to outbreaks identified by the IPC department. For clusters that were characterized with molecular data, MRSA cases that comprised significant clusters were evaluated based on *spa* type. Characteristics of significant clusters (e.g., time frame, observed and expected case numbers, P-value, and *spa* type) are reported.

## Results

### Descriptive statistics

Data on 735 MRSA cases, from 686 patients, were obtained during the study period. A total of 642 (93.6%) patients were identified with MRSA during one hospital admission-discharge period and 44 (6.4%) patients were identified with MRSA during two or more hospital admission-discharge periods. For the 686 patients, 51.6% (n = 354) were male and 48.4% (n = 332) were female. For male patients, ages ranged from 21 months to 97 years (mean = 72.6 years) and for female patients, ages ranged from 7 to 100 years (mean = 73.8 years).

**Figure 1:**
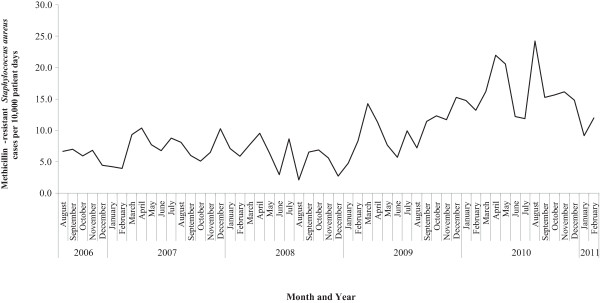
**Incidence rate of methicillin-resistant *Staphylococcus aureus *** **infections and colonization per month in a community hospital.**

The monthly incidence rate of MRSA fluctuated over the study period (Figure 1) ranging from 2.10 to 24.22 MRSA cases (colonization and infection)/10,000 patient days with a mean of 9.59 MRSA cases/10,000 patient days. Summary characteristics of the MRSA incidence rate per month, year, season, service, and ward are presented in Table 1. Overall, the highest incidence rates of MRSA occurred during 2010 and in 2011 (during the first two months in which surveillance data were available). On average, March, April, and May reported the highest MRSA rates on a monthly basis. At the service level, the highest MRSA rates occurred in the surgery, internal medicine, and hospice departments, whereas, the pediatric department reported the lowest MRSA incidence rate in the hospital.

**Table 1:** Summary characteristics of 735 cases of MRSA, August 1, 2006 - February 28, 2011

**MRSA Characteristics**	**Incidence rate of MRSA ** **(cases per 10,000 patient days)**
Month^1^	
January	7.99
February	8.66
March	11.87
April	13.29
May	10.58
June	6.90
July	9.79
August	9.65
September	9.24
October	9.17
November	9.34
December	9.49
Year^2^	
2006 (August – December)	6.14
2007 (January – December)	7.28
2008 (January – December)	5.97
2009 (January – December)	9.98
2010 (January – December)	16.42
2011 (January – February)	10.49
Season^1^	
Spring (April – June)	10.26
Summer (July – September)	9.56
Fall (October – December)	9.33
Winter (January – March)	9.50
Service^3^	
Acute care	10.12
Complex care	9.09
Hospice	14.11
Intensive care unit	9.00
Internal medicine	15.11
Oncology	3.90
Pediatrics	0.22
Psychiatry	6.08
Rehabilitation	8.57
Surgery	17.41
Ward^3^	
S1 (Surgery)	19.39
S2 (Surgery)	15.14
M1 (Internal medicine)	23.14
C1 (Complex continuing care)	9.15
C2 (Complex continuing care)	8.78

MRSA = Methicillin-resistant *Staphylococcus aureus.*

^1^Incidence rate presented is based on an average for that specific period.

^2^Incidence rate presented is the total for that specific year.

^3^Incidence rate presented is the total for that service or ward from August 1, 2006 to February 28, 2011.

From March 1, 2010 to February 28, 2011, 267 MRSA cases were identified and 167 (62.5%) patient isolates were obtained for *spa* typing (Table 2). Overall, nine different *spa* types were identified with *spa* type 2/t002 (88.6%) the most prevalent. When *spa* types were categorized according to the epidemic CMRSA type, the majority (97.6%) of *spa* types were classified as CMRSA-2 (Table 2). Only one *spa* type, 93/t026, was classified as CMRSA-1. All isolates were negative for the PVL toxin gene.

**Table 2:** Typing data for 167 MRSA patient isolates

**eGenomics *spa *** **type**^**1**^	**Number of MRSA isolates (%)**	**Ridom *spa *** **type**^**2**^	**PVL gene**	**CMRSA**
2	148 (88.6)	t002	No	2
23	3 (1.8)	t548	No	2
26	1 (0.6)	t539	No	2
93	4 (2.4)	t026	No	1
228	1 (0.6)	t5518	No	2
230	1 (0.6)	t010	No	2
268	4 (2.4)	t067	No	2
387	4 (2.4)	t653	No	2
1178	1 (0.6)	t5607	No	2

MRSA = Methicillin-resistant *Staphylococcus aureus.*

PVL = Panton-Valentine leukocidin.

CMRSA = Canadian epidemic methicillin-resistant *Staphylococcus aureus.*

^1^*spa* types classified according to eGenomics (http://tools.egenomics.com).

^2^*spa* types classified according to the Ridom system (http://www.spaserver.ridom.de).

**Figure 2:**
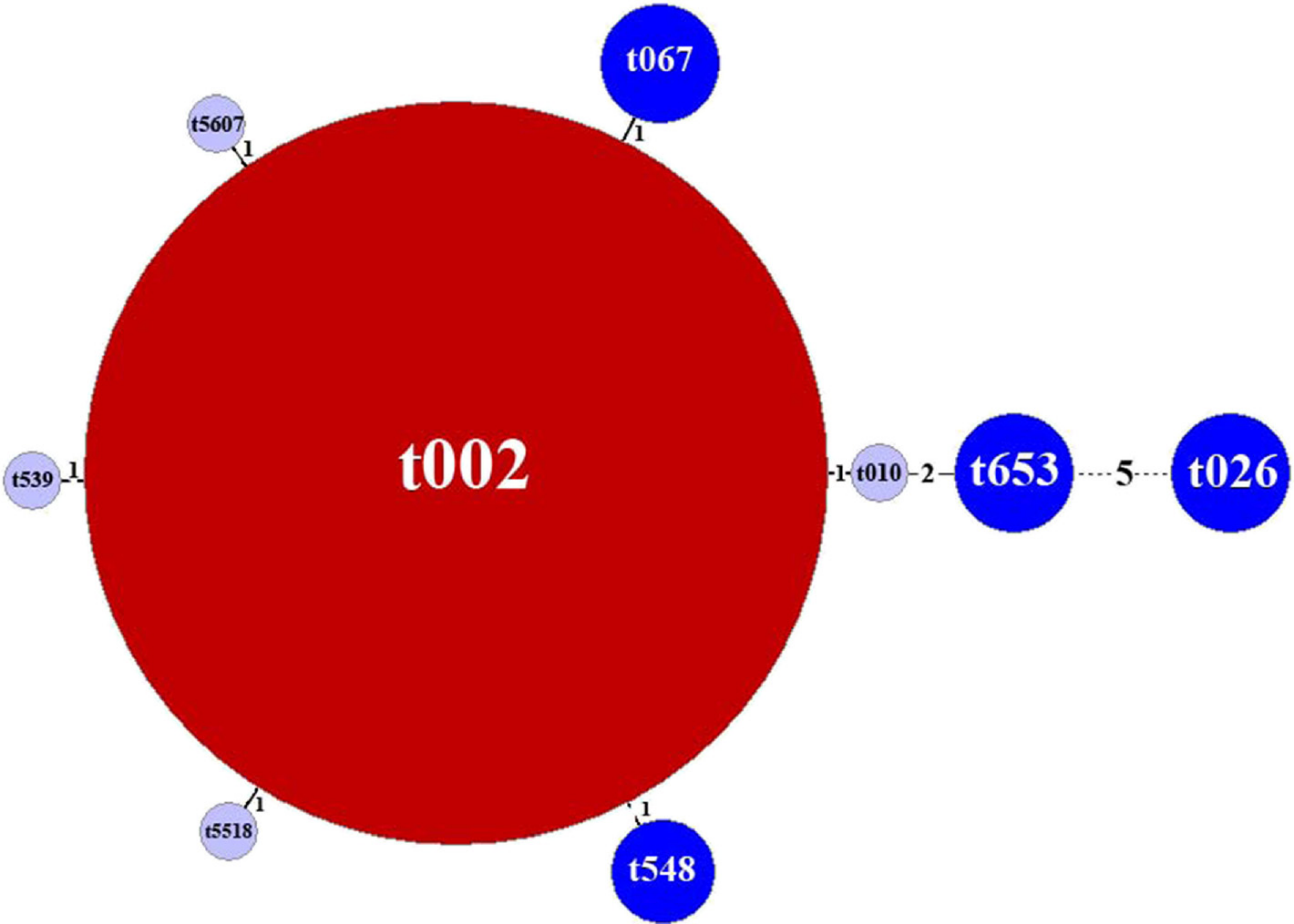
**Minimum spanning tree analysis of 167 methicillin-resistant *Staphylococcus aureus *(MRSA) patient isolates.** The size of each circle is proportional to the number of isolates. Ridom *spa* types are presented in each circle. Numbers intersecting the black lines indicate the difference in the number of repeats between connecting circles. Colours refer to the number of MRSA isolates: red > 20, dark blue ≤ 5, light blue ≤ 2.

Based on the MST that was constructed for clustering *spa* types (Figure 2), one major group was observed with seven different *spa* types reported as being closely related (i.e., difference is ≤ 2 repeats) to *spa* type 2/t002. These closely related *spa* types all corresponded to CMRSA-2.

For two or more MRSA isolates found with identical *spa* types, antimicrobial susceptibility profiles of *spa* types were examined to determine if the molecular findings could be further characterized (Table 3). For *spa* types 23/t548, 93/t026, 268/t067, and 387/t653, the antimicrobial susceptibility profiles were identical between *spa* types. For *spa* type 2/t002, three different antimicrobial susceptibility profiles were identified.

**Table 3:** **Antimicrobial susceptibility profiles, based on *spa *** type, from 149* MRSA patient isolates

***spa *** **type**	**Number of MRSA isolates**	**Rifampin**	**Clindamycin**	**Trimethoprim and sulfamethoxazole**	**Vancomycin**
2	124	Susceptible	Resistant	Susceptible	Susceptible
2	1	Susceptible	Resistant	Resistant	Susceptible
2	9	Susceptible	Susceptible	Susceptible	Susceptible
23	3	Susceptible	Resistant	Susceptible	Susceptible
93	4	Susceptible	Resistant	Susceptible	Susceptible
268	4	Susceptible	Resistant	Susceptible	Susceptible
387	4	Susceptible	Resistant	Susceptible	Susceptible

*Antimicrobial susceptibility profiles are only presented for *spa* types in which ≥ 2 MRSA isolates were identified.

MRSA = Methicillin-resistant *Staphylococcus aureus.*

### Statistical model

A random effects negative binomial model was chosen over the random effects Poisson model based on the AIC value. Results from the univariable multilevel negative binomial models indicated that year and month were all significantly associated with the rate of MRSA cases in the hospital [see Additional File 1]. The final multivariable multilevel negative binomial model indicated that year and month were significantly associated with the rate of MRSA cases (Table 4). Interactions between the variables year and month could not be assessed due to the number of categories for each independent variable and the resulting small number of observations per interaction term. For the independent variable year, years 2009–2011 had MRSA rates that were significantly higher than the MRSA rates in 2006. Results from model-based contrasts indicate that years 2009–2011 also had significantly higher MRSA rates than years 2007 and 2008 (Table 5). In the final model, the rates of MRSA were also significantly higher in months March and April compared to January. Results from model-based contrasts demonstrated that significant increased MRSA rates were also noted in March and April compared to June, and for April compared to February.

**Table 4:** Multivariable multilevel* negative binomial regression model of variables associated with the rate of MRSA cases

**Variable**	**Description**	**IRR**	**95% CI**	**P-value**
Year	2006	Referent		
	2007	1.12	0.73 – 1.72	0.607
	2008	0.89	0.57 – 1.39	0.611
	2009	1.63	1.08 – 2.46	0.021
	2010	2.62	1.76 – 3.89	< 0.001
	2011	2.14	1.13 – 4.06	0.020
Month	January	Referent		
	February	1.09	0.70 – 1.71	0.689
	March	1.63	1.04 – 2.57	0.035
	April	1.88	1.22 – 2.91	0.005
	May	1.33	0.83 – 2.15	0.234
	June	0.89	0.53 – 1.51	0.664
	July	1.27	0.79 – 1.06	0.320
	August	1.44	0.92 – 2.26	0.111
	September	1.37	0.87 – 2.17	0.177
	October	1.33	0.84 – 2.10	0.221
	November	1.34	0.85 – 2.12	0.207
	December	1.42	0.91 – 2.23	0.122
Service	Medicine^1^	Referent		
	Surgery	0.95	0.30 – 2.96	0.924
	Other^2^	0.42	0.17 – 1.05	0.064

^*^Random intercept for ward.

MRSA = Methicillin-resistant *Staphylococcus aureus.*

IRR = Incidence rate ratio.

CI = Confidence interval.

^1^Included the following departments: intensive care (adult and neonatal), oncology, pediatrics, and internal medicine.

^2^Included the following departments: psychiatry, rehabilitation, hospice, childbirth, and nursery.

**Table 5:** Significant model-based contrasts between the rate of MRSA and year and month

**Description**	**IRR**	**95% CI**	**P-value**
2009 versus 2007	1.45	1.09 – 1.94	0.011
2009 versus 2008	1.83	1.34 – 2.48	< 0.001
2010 versus 2007	2.34	1.79 – 3.06	< 0.001
2010 versus 2008	2.94	2.20 – 3.92	< 0.001
2010 versus 2009	1.61	1.28 – 2.03	< 0.001
2011 versus 2007	1.91	1.10 – 3.31	0.021
2011 versus 2008	2.39	1.38 – 4.18	0.002
March versus June	1.83	1.12 – 3.00	0.016
April versus February	1.72	1.11 – 2.66	0.015
April versus June	2.11	1.31 – 3.42	0.002

MRSA = Methicillin-resistant *Staphylococcus aureus.*

IRR = Incidence rate ratio.

CI = Confidence interval.

### Temporal scan statistic

Over the study period, the temporal scan statistic identified statistically significant MRSA clusters at the hospital (n = 2), service (n = 16), and ward (n = 10) levels (Table 6). As separate scan tests were performed at various levels, it was observed that several clusters overlapped in time and location (i.e., service and wards). Of the 26 clusters identified at the service and ward levels, 15 were classified as unique events.

**Table 6:** Statistically significant temporal clusters of MRSA rates, August 1, 2006 - February 28, 2011

**Cluster Number**	**Cluster Description**	**Date of cluster (year/month/day)**	**Number of MRSA cases**	**Observed/Expected**	**P-value**	***spa *** **type (n)**
**Hospital wide scans**						
1	2006-2011^1^	2009/9/1 – 2011/2/28	376	1.55	< 0.001	2(148), 23(3), 26(1), 93(4), 228 (1), 230 (1), 268(4), 387(4), 1178(1)
2	2006-2011^2,3^	2009/3/1 – 2009/4/30	37	1.84	0.038	Not applicable
**Service scans**						
3	Acute care, 2006-2011^3^	2010/3/1 – 2010/6/30	6	7.44	0.007	2(1)
4^a^	Complex care, 2006-2011^1^	2009/2/1 – 2011/1/31	130	1.5	< 0.001	2(43), 23(2), 228(1), 268(1)
5	Intensive care unit, 2006-2011^2,3^	2010/6/1 – 2010/11/30	13	3.06	0.022	2(7), 268(1)
6	Intensive care unit, 2006-2011^4^	2007/10/1 – 2008/1/31	8	4.1	0.038	Not applicable
7^b^	Internal medicine, 2006-2011^1^	2009/10/1 - 2010/12/31	95	2.0	< 0.001	2(37), 93(2), 387(1)
8	Oncology, 2006-2011^1^	2009/12/1 – 2010/12/31	11	3.42	0.002	2(5)
9^c^	Psychiatry, 2006-2011^1^	2006/9/1 – 2008/5/31	47	2.36	< 0.001	Not applicable
10^c^	Psychiatry, 2008^3^	2008/1/1 – 2008/5/31	9	2.16	0.025	Not applicable
11	Rehabilitation, 2006-2011^2,3,5^	2008/7/1 – 2008/7/31	6	6.68	0.035	Not applicable
12	Rehabilitation, 2006-2011^1^	2009/2/1 – 2011/2/28	32	1.57	0.038	2(9), 26(1), 93(1), 268(1)
13^d^	Surgery, 2006-2011^1,4^	2009/9/1 – 2011/2/28	106	1.74	< 0.001	2(40), 23(1), 93(1), 230(1), 268(1), 387(2)
14^e^	Surgery, 2006-2011^1,4^	2008/9/1 – 2009/3/31	31	2.08	0.003	Not applicable
15	Surgery, 2007^2,3^	2007/3/1 – 2007/4/30	9	2.83	0.026	Not applicable
16	Surgery, 2007^3^	2007/8/1 – 2007/10/31	8	3.19	0.002	Not applicable
17^f^	Surgery, 2008^2,3,4^	2008/9/1 – 2008/11/30	18	2.25	0.003	Not applicable
18^g^	Surgery, 2008^2,3,4^	2008/3/1 – 2008/5/31	12	2.32	0.004	Not applicable
**Ward scans**						
19^b^	M1 (Internal medicine), 2006-2011^4^	2010/1/1 – 2010/5/31	26	2.29	0.009	2(6), 93(1)
20^d^	S1 (Surgery), 2006-2011^4^	2009/7/1 – 2011/2/28	70	1.74	< 0.001	2(23), 23(1), 93(1), 268(1), 387(2)
21^e^	S1 (Surgery), 2006-2011^1^	2008/4/1 – 2009/3/31	29	1.96	0.001	Not applicable
22^f^	S1 (Surgery), 2008^4^	2008/10/1 – 2008/10/31	8	3.91	0.010	Not applicable
23^g^	S1 (Surgery), 2008^4^	2008/4/1 – 2008/5/31	10	3.16	0.003	Not applicable
24^d^	S2 (Surgery), 2006-2011^4^	2009/2/1 – 2011/2/28	52	1.56	0.002	2(17), 230(1)
25^e^	S2 (Surgery), 2008^4^	2008/9/1 – 2008/11/30	7	3.25	0.024	Not applicable
26^a^	C2 (Complex care), 2006-2011^1^	2010/4/1 – 2011/2/28	27	3.09	< 0.001	2(19), 268(1), 1178(1)
27^a^	C2 (Complex care), 2010^2,3^	2010/8/1 – 2010/11/30	17	2.04	0.009	2(14), 268(1)
28^a^	C1 (Complex care), 2009^3^	2009/4/1 – 2009/9/30	11	1.84	0.031	Not applicable

MRSA = Methicillin-resistant *Staphylococcus aureus.*

n = Number of isolates.

^1^Long duration cluster (7–25 months in length).

^2^Clusters are potential outbreaks.

^3^Short duration cluster (1–6 months in length).

^4^Cluster was identified as part of a MRSA outbreak identified by Infection Prevention and Control personnel.

^5^Cluster was also identified in the Rehabilitation department for the 2008 annual analysis.

^a-g^Indicates a cluster identified by >1 temporal scan at the service and ward levels.

Overall, clusters ranged in duration from 1 to 25 months (mean = 9.1 months) and involved 6 to 376 patients (mean = 43.3) per cluster. Using routine surveillance, IPC personnel identified nine MRSA outbreaks during the study period. These outbreaks occurred in five wards (equivalent to three services), ranged from 2 weeks to 14 months in duration, and involved 4 to 68 patients (mean = 14) per outbreak. Seven (77.8%) of these previously known outbreaks were identified as significant clusters (Cluster ID 6, 19, 20, 22–25) based on the temporal scan statistic. Of the seven events that were identified by both IPC personnel and the temporal scan statistic, three clusters (Cluster ID 19, 20, 24) were further characterized using molecular data. For all three clusters, more than one *spa* type was identified; however, *spa* type 2/t002 was the most prevalent MRSA strain in all three events. For the cluster identified in ward S1 from July 2009 to February 2011, further investigation revealed that *spa* type 387/t653 was identified in two different patients in November, 2010.

For the remaining MRSA clusters, 11 (52.4%) events were considered to be of short duration (i.e., 1–6 months in length) and 10 (47.6%) events were considered to be of long duration (i.e., 7–25 months in length). Examination of short duration clusters revealed that seven of the events (Cluster ID 2, 5, 11, 15, 17, 18, 27) may have been potential outbreaks as there were considerably large numbers of MRSA cases detected during a short time span. For two of these clusters, typing data indicated that *spa* type 2/t002 was the prevalent MRSA strain identified in the patient population.

For 10 long duration clusters, seven (Cluster ID 1, 4, 7, 8, 12, 13, 26) were further characterized with molecular data. For six of these events (Cluster ID 1, 4, 7, 12, 13, 26), several *spa* types were identified circulating in the patient population. However, like the short duration clusters, *spa* type 2/t002 was the predominant MRSA strain in all seven events. Further analysis of the long duration clusters revealed potential transmission of non-*spa* type 2 MRSA strains. For the complex care cluster (Cluster ID 4), *spa* type 23/t548 was identified in two different patients, both admitted to the same ward, in December 2010. In the internal medicine cluster (Cluster ID 7), two patients were identified with *spa* type 93/t026, seven weeks apart. For the surgery cluster (Cluster ID 13), two patients, located in the same ward, were identified with *spa* type 387/t653 in November 2010.

## Discussion

In the present investigation, a cluster was defined as a statistically significant high rate of MRSA cases within a time period. Using the temporal scan statistic, several significant MRSA clusters were identified, during the 2006–2011 surveillance period, in a community hospital. During the same time period, nine MRSA outbreaks were identified by IPC personnel using standardized surveillance techniques. However, only seven of these outbreaks were identified by the temporal scan statistic as significant clusters. This was not surprising, as others have reported similar results regarding the discordance between events identified by hospital staff and the identification of clusters employing a scan statistic [2,4].

Investigation of the seven outbreaks identified by both hospital staff and the temporal scan statistic indicated that five (Cluster ID 19, 20, 23–25) were identified by the scan test with a starting and/or end date that were different from the dates provided by IPC personnel. Specifically, clusters identified by the temporal scan statistic were one to seven months longer in duration; consequently, a greater number of MRSA cases per cluster were identified. For four of the clusters (Cluster ID 19, 23–25), the temporal scan statistic indicated that these events occurred prior to dates reported by IPC personnel. This is an important finding as these events demonstrate that there may have been a delay in the institution of infection control strategies. Furthermore, two of the clusters (Cluster ID 20, 24) were identified as being longer in duration by the temporal scan statistic; therefore, premature discontinuation of infection control measures by IPC personnel may have occurred.

For the two MRSA outbreaks identified by IPC staff only, one outbreak was located in the intensive care unit (n = 6 patients, 1 month in duration) and the other outbreak was located in a ward in the internal medicine department (n = 11 patients, 6 weeks duration). The outbreak in the intensive care unit was identified by the temporal scan statistic; however, the cluster was not significant. For the outbreak identified in the ward of the internal medicine department, this particular ward was only operational for 27 of the 55 months that the study was conducted, and therefore, did not meet the inclusion criteria for a temporal scan test to be conducted at the ward level. However, temporal scan tests conducted at the hospital and service (e.g., internal medicine) levels did not result in the identification of a cluster that corresponded in time to the MRSA outbreak. In the participating healthcare facility, specific rule-based criteria (e.g., ≥ 3 HA-MRSA cases in a 2 week period) are not used to establish MRSA outbreaks. Rather, data pertaining to the number of MRSA cases and the time period (i.e., number of days) are used to ascertain if an outbreak exists in the patient population and if an investigation should be initiated. This type of surveillance is subjective in nature and may be prone to under-reporting or over-reporting of MRSA outbreaks.

Of the 11 short duration (e.g., 1–6 months) clusters that were identified during this investigation, seven events were labelled by the investigators as potential outbreaks due to the large number of MRSA cases identified over a short time period. However, only two of these events could be analyzed at the molecular level, with *spa* type 2/t002 as the predominant MRSA strain in each cluster. These molecular findings indicate that transmission events may have occurred and that these clusters may have been equivalent to true outbreaks.

For the 10 long duration (e.g., 7–25 months) clusters identified, their biological relevance is difficult to discern. These clusters may represent extended outbreaks, temporal trends, changes in pathogen characteristics, or the representation of systematic changes (e.g., cleaning policies) at the hospital level during the surveillance period. For many of the long duration clusters, several non-*spa* type 2/t002 MRSA strains were identified in the patient population. These particular MRSA strains may indicate unique transmission events between patients or be part of an outbreak, as researchers have documented the existence of more than one MRSA strain during an outbreak investigation [25,26]. Alternatively, these non-*spa* type 2/t002 strains may represent genetic changes within the *spa* gene, such as deletions or duplications of repeats and point mutations [27], resulting in different *spa* types. Based on the MST that was constructed (Figure 2), six of the *spa* complexes were closely related to *spa* type 2/t002 by a difference in one repeat. For MRSA and *S. aureus*, research exploring the time required for a genetic event to occur has been conducted [28-30]; however, results of these investigations are based on specific strains and specific locations of the genetic event. Other plausible explanations for observing diverse *spa* types in these clusters include the introduction of *spa* types into the hospital via staff, visitors, and patients which resulted in transmission events, and healthcare workers and patients that may have been colonized with variant MRSA strains.

In the study hospital, molecular typing of MRSA isolates is not routinely performed. This was a major limitation of this study, as there was no prior knowledge of the endemic MRSA strains in this facility. Furthermore, not all MRSA isolates were collected from the hospital’s microbiology laboratory for *spa* typing. Consequently, the true molecular composition for some clusters is not known, and it could not be determined if all cases within clusters were a result of unique transmission events or part of a true outbreak. Lastly, with the identification of a predominant *spa* type circulating in the patient population, the application of *spa* typing provided very little benefit for elucidating transmission events or recognizing potential outbreaks, especially for long duration clusters. Although it was not conducted in this study, the incorporation of whole-genome sequencing may be a viable tool for further elucidating possible transmission events and identify potential/true outbreaks in the hospital setting. Whole-genome sequencing provides an inventory of the microevolutionary changes of a bacterium and can be used to map genome-wide single-nucleotide polymorphisms, insertions, and deletions to a reference sequence [28]. Furthermore, this typing technique provides the best discrimination between closely related bacterial isolates in a timely manner [1,29].

For *spa* types identified with ≥ 2 MRSA isolates, antimicrobial susceptibility profiles were examined to determine if differences in antibiograms could provide further characterization of clusters. For *spa* type 2/t002, nine MRSA isolates were identified as being susceptible to clindamycin whereas all other *spa* type 2/t002 isolates were resistant. Further investigation of these nine MRSA isolates revealed that three isolates were identified in the same internal medicine cluster (Cluster ID 7; n = 2 in May, 2010; n = 1 in December, 2010) and three isolates were identified in the same surgery cluster (Cluster ID 13; n = 1 in July, 2010; n = 1 in August, 2010; n = 1 in September, 2010). The identification of identical *spa* types and antibiograms in two long duration clusters, suggests that transmission events among patients, hospital staff, or possibly even the contaminated environment, may have occurred. For *spa* types 23/t548, 93/t026, 268/t067, and 387/t653, it was noted that these particular *spa* types had indistinguishable antimicrobial profiles. This demonstrates that for the identification of transmission events and the determination of cases as outbreak or non-outbreak, relying on antibiogram data may result in inaccurate findings and the misclassification of cases as MRSA strains that differ genotypically may display identical antimicrobial susceptibility profiles.

During the study period, the incidence rate of MRSA fluctuated considerably, with significant increases noted in years 2009–2011. Starting in December 2008, Ontario hospitals were required to report the number of newly acquired HA-MRSA bacteremias on a quarterly basis, to be posted on a web site that is accessible to the public [31]. Although the figures are affiliated with bacteremias only, the public reporting of MRSA did not result in a dramatic decrease in the overall incidence of MRSA in this facility, which is in contrast to *C. difficile*. In September 2008, monthly data on *C. difficile* infections (CDIs) from Ontario hospitals were also posted on a publicly accessed web site [32]. At the participating hospital, a separate analysis of CDIs demonstrated that in 2006 and 2007, there were significantly more cases of CDI, compared to 2008 and 2009 (data not shown). It is theorized that the decrease in *C. difficile* case rates may be attributable to hospitals adhering to best practices in *C. difficile* prevention due to the mandatory public reporting of rates [33]. It was anticipated that this would also apply to MRSA; however, this was not observed.

The significant increases in the incidence rate of MRSA cases in years 2009–2011, based on the final multivariable model and model-based contrasts, are concordant with the findings from the temporal scan statistic as approximately 60% of the clusters identified spanned 2009–2011. The increase in the MRSA case rate during this time period may have been due to the presence of respiratory viruses (e.g., influenza) within the hospital. Reports of increased hospital MRSA rates during outbreaks of respiratory pathogens have been published [34,35]. In the northern hemisphere, influenza season occurs from October to March [36]. For 2009–2010, in addition to the regular influenza season, the H1N1 influenza pandemic was identified in Canada [37] and H1N1 patients were admitted to the participating facility. An increase in the MRSA case rate may have occurred as infection control activities and surveillance were focused on influenza and away from MRSA [34], reduced staffing as a result of illness, antimicrobial prescribing practices [35] especially with fluoroquinolones which may have led to an increase in the risk for acquiring MRSA [38], and environmental contamination of MRSA which may have resulted in transmission events among staff and patients.

The independent variable season was not found to be significantly associated with the number of MRSA cases in this hospital. Seasonal variation in MRSA is debatable with some studies documenting spring [39] or summer [40,41] with increased MRSA rates. However, in these studies, specific aspects of MRSA infections (e.g., community-acquired, healthcare-acquired, severe only) were investigated. A significant increase in the rate of MRSA cases was observed specifically in the months of March and April. As previously discussed, an increase in the number of MRSA cases in March may have been a result of the presence of influenza, or other respiratory viruses, in the hospital.

## Conclusions

The application of a temporal scan statistic to historical data from a community hospital resulted in the identification of several significant MRSA clusters. Further examination of these clusters revealed several events that may be equivalent to MRSA outbreaks or transmission events that were not recognized by hospital personnel. By adopting a comprehensive approach for MRSA surveillance, clusters were identified at the hospital, service, and ward levels. Infection control efforts can be focused at one or more levels to identify risk factors for MRSA acquisition and transmission, establish interventions, and evaluate control measures. The identification of specific time periods that corresponded to significant increases in the rate of MRSA cases in the patient population may have been correlated with other determinants at the hospital level, including the presence of other pathogens. In this investigation, *spa* typing provided very little information due to the presence of a predominant *spa* type. Therefore, the use of a different typing technique (e.g., whole-genome sequencing) or additional supplementary information may be warranted to decipher transmission events and clusters. Application of scan statistics for hospital surveillance of MRSA would probably be most rewarding in facilities with access to higher resolution molecular typing data. Future research utilizing the temporal scan statistic, prospectively, with the application of molecular typing, especially whole-genome sequencing, to identify MRSA clusters in real-time and elucidate transmission events in the hospital setting, is warranted.

## Abbreviations

AIC: Akaike’s Information Criteria; CDIs: *Clostridium difficile* infections; CI: Confidence interval; CMRSA: Canadian epidemic methicillin-resistant *Staphylococcus aureus*; HA: Healthcare-associated; HAIs: Healthcare-associated infections; IPC: Infection Prevention and Control; IRR: Incidence rate ratio; MRSA: Methicillin-resistant *Staphylococcus aureus*; MST: Minimum spanning tree; n: Number of isolates; PVL: Panton-Valentine leukocidin; ST: Sequence type.

## Competing interests

The authors declare that they have no competing interests.

## Authors’ contributions

MCF contributed to the design of the study, statistical and molecular analyses, and drafting of the manuscript. DLP and OB contributed to study design and statistical analysis. WAC contributed to study design and data collection. RRS contributed to study design. JSW contributed to study design and molecular analysis. All authors contributed to the editing and final version of the manuscript.

## Pre-publication history

The pre-publication history for this paper can be accessed here:

http://www.biomedcentral.com/1471-2334/14/375/prepub

## Supplementary Material

Additional file 1 Univariable multilevel* negative binomial regression analyses of variables associated with the rate of MRSA cases. Click here for file
