# Supplementary material for: The use of the temporal scan statistic to detect methicillin-resistant Staphylococcus aureus clusters in a community hospital
Source: BMC Infect Dis. 2014 Jul 8;14:375. doi: 10.1186/1471-2334-14-375 (PMC4097048; doi:10.1186/1471-2334-14-375)
Supplement: Additional file 1 — Univariable multilevel* negative binomial regression analyses of variables associated with the rate of MRSA cases. [file 1471-2334-14-375-S1.doc]

Additional File 1: Univariable multilevel* negative binomial regression analyses of variables associated with the rate of MRSA cases.

| **Variable** | **Description** | **IRR** | **95% CI** | **P-value** |
| --- | --- | --- | --- | --- |
| Year | 2006 | Referent |  |  |
|  | 2007 | 1.10 | 0.72 – 1.68 | 0.651 |
|  | 2008 | 0.85 | 0.55 – 1.32 | 0.470 |
|  | 2009 | 1.52 | 1.01 – 2.28 | 0.043 |
|  | 2010 | 2.45 | 1.65 – 3.62 | < 0.001 |
|  | 2011 | 1.59 | 0.89 – 2.81 | 0.115 |
| Season | Fall | Referent |  |  |
|  | Winter | 1.01 | 0.77 – 1.33 | 0.933 |
|  | Spring | 1.05 | 0.79 – 1.39 | 0.729 |
|  | Summer | 1.02 | 0.78 – 1.35 | 0.868 |
| Month | January | Referent |  |  |
|  | February | 1.14 | 0.69 – 1.86 | 0.612 |
|  | March | 1.62 | 1.01 – 2.61 | 0.045 |
|  | April | 1.78 | 1.12 – 2.84 | 0.015 |
|  | May | 1.19 | 0.71 – 1.99 | 0.520 |
|  | June | 0.87 | 0.50 – 1.52 | 0.628 |
|  | July | 1.29 | 0.79 – 2.14 | 0.305 |
|  | August | 1.17 | 0.72 – 1.91 | 0.524 |
|  | September | 1.23 | 0.76 – 1.99 | 0.392 |
|  | October | 1.16 | 0.71 – 1.89 | 0.558 |
|  | November | 1.18 | 0.73 – 1.92 | 0.497 |
|  | December | 1.27 | 0.79 – 2.03 | 0.328 |
| Service | Medicine^1^ | Referent |  |  |
|  | Surgery | 0.86 | 0.40 – 1.86 | 0.706 |
|  | Other^2^ | 0.92 | 0.44 – 1.93 | 0.825 |

^*^ Random intercept for ward

MRSA = Methicillin-resistant *Staphylococcus aureus*

IRR = Incidence rate ratio

CI = Confidence interval

^1^ Included the following departments: intensive care (adult and neonatal), oncology, pediatrics, and internal medicine

^2^ Included the following departments: psychiatry, rehabilitation, hospice, childbirth, and nursery
